# Deep learning-based prediction for significant coronary artery stenosis on coronary computed tomography angiography in asymptomatic populations

**DOI:** 10.3389/fcvm.2023.1167468

**Published:** 2023-06-21

**Authors:** Heesun Lee, Bong Gyun Kang, Jeonghee Jo, Hyo Eun Park, Sungroh Yoon, Su-Yeon Choi, Min Joo Kim

**Affiliations:** ^1^ Department of Internal Medicine, School of Medicine, Seoul National University, Seoul National University Hospital Healthcare System Gangnam Center, Seoul, Republic of Korea; ^2^ Interdisciplinary Program in Artificial Intelligence, Seoul National University, Seoul, Republic of Korea; ^3^ Institute of New Media and Communications, Seoul National University, Seoul, Republic of Korea; ^4^ Department of Electrical and Computer Engineering, Seoul National University, Seoul, Republic of Korea

**Keywords:** coronary artery disease, coronary stenosis, computed tomographic angiography, deep learning, neural networks, diagnostic screening programs

## Abstract

**Background:**

Although coronary computed tomography angiography (CCTA) is currently utilized as the frontline test to accurately diagnose coronary artery disease (CAD) in clinical practice, there are still debates regarding its use as a screening tool for the asymptomatic population. Using deep learning (DL), we sought to develop a prediction model for significant coronary artery stenosis on CCTA and identify the individuals who would benefit from undergoing CCTA among apparently healthy asymptomatic adults.

**Methods:**

We retrospectively reviewed 11,180 individuals who underwent CCTA as part of routine health check-ups between 2012 and 2019. The main outcome was the presence of coronary artery stenosis of ≥70% on CCTA. We developed a prediction model using machine learning (ML), including DL. Its performance was compared with pretest probabilities, including the pooled cohort equation (PCE), CAD consortium, and updated Diamond-Forrester (UDF) scores.

**Results:**

In the cohort of 11,180 apparently healthy asymptomatic individuals (mean age 56.1 years; men 69.8%), 516 (4.6%) presented with significant coronary artery stenosis on CCTA. Among the ML methods employed, a neural network with multi-task learning (19 selected features), one of the DL methods, was selected due to its superior performance, with an area under the curve (AUC) of 0.782 and a high diagnostic accuracy of 71.6%. Our DL-based model demonstrated a better prediction than the PCE (AUC, 0.719), CAD consortium score (AUC, 0.696), and UDF score (AUC, 0.705). Age, sex, HbA1c, and HDL cholesterol were highly ranked features. Personal education and monthly income levels were also included as important features of the model.

**Conclusion:**

We successfully developed the neural network with multi-task learning for the detection of CCTA-derived stenosis of ≥70% in asymptomatic populations. Our findings suggest that this model may provide more precise indications for the use of CCTA as a screening tool to identify individuals at a higher risk, even in asymptomatic populations, in clinical practice.

## 1. Introduction

Coronary heart disease (CHD) is a leading cause of morbidity and mortality worldwide, contributing to one-third of global deaths (1, 2). Since the treatment of CHD causes a considerable medical and socioeconomic burden, recent clinical interests have been focused on risk stratification, early detection, and prevention of the disease (3, 4). Although traditional risk classification tools, such as Pooled Cohort Equation (PCE) or Systemic Coronary Risk Estimation, have been a priority to estimate the risk of CHD (3, 4), these are imprecise and less practical, and may result in unnecessary long-term therapies or loss of opportunity for timely management (5). Recently, coronary computed tomography angiography (CCTA) has emerged as the frontline test to noninvasively evaluate CHD, providing excellent diagnostic accuracy and prognostic implication (6, 7). The SCOT-HEART investigators demonstrated that CCTA-guided preventive therapy could bring a significant reduction in cardiovascular death or nonfatal myocardial infarction in patients with stable chest pain (7). Indeed, the advent of CCTA can bring a paradigm shift in this field (8). On the other hand, there is still debate in the screening of CHD using CCTA among apparently healthy asymptomatic populations, because of unclear superiority to traditional approach and lack of cost-effectiveness (4, 9). Data from large-scale cohorts consistently reported that the prevalence of occult coronary atherosclerosis was not negligible in the asymptomatic populations, with approximately 5% of them having significant stenosis (10, 11). In addition, as the prognostic value of CCTA was well validated in this population (12, 13), some experts state that CCTA has the potential as a good screening tool for asymptomatic individuals (9, 14). Considering the expansion of indications for CCTA in clinical practice, it is required to identify which patient would be beneficial from CCTA.

The development of deep learning (DL) has recently achieved professional-level performance in various clinical data analyses, such as medical imaging and electronic health records, and has attracted much attention in the field of medical diagnosis (15–17). As DL models consist of a large number of parameters compared to statistical methods, this complexity enables the ability to express complex correlations between variables and provide meaningful insights for better medical decision-making. In particular, DL methods have been increasingly applied in medical imaging interpretation with reliable results (18, 19), and also in prediction model development (17). Hence, we sought to develop a DL-based risk prediction model for coronary artery stenosis on CCTA in apparently healthy asymptomatic adults and identify the beneficiaries for introducing CCTA as a screening tool.

## 2. Materials and methods

### 2.1. Study population and dataset composition

**Figure 1:**
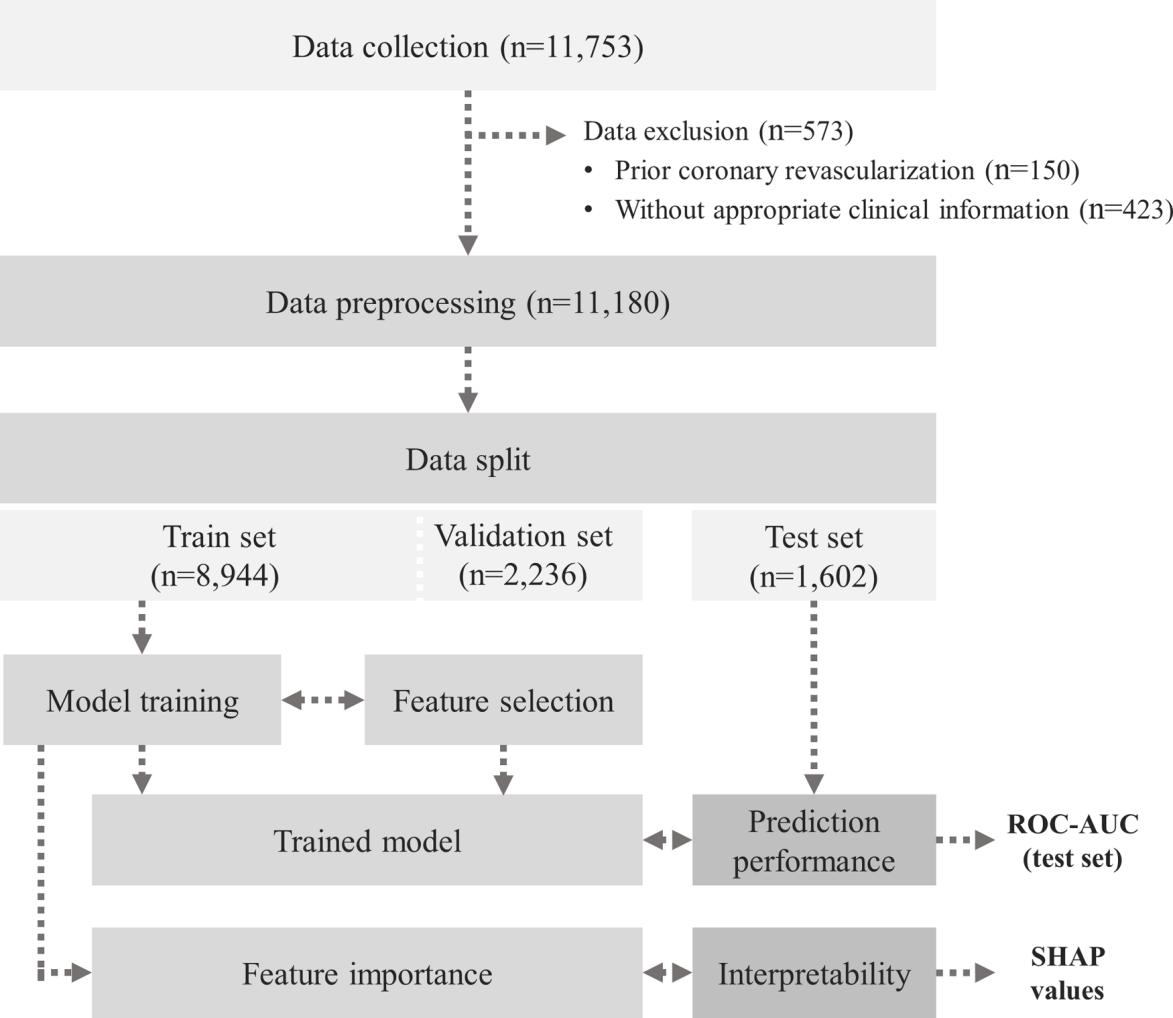
Schematic flowchart of the study population. AUC, area under the curve; ROC, receiver operating characteristic; SHAP, SHapley Additive ExPlanations.

A total of 11,753 medical records were retrospectively collected from individuals who underwent CCTA for the purpose of health check-ups at the Healthcare System Gangnam Centre, Seoul National University Hospital, between January 2012 and December 2019. The individuals chose to undergo the examinations for health status evaluation of their own will. If an individual had symptoms, he/she was recommended to visit the corresponding outpatient clinic rather than a health check-up. Clinical and laboratory information of the study participants were retrieved from their examination results, which were performed on the same day as the CCTA. Individuals with prior coronary revascularization (*n* = 150) and those without appropriate clinical information (*n* = 423) were excluded from the analysis. Finally, we established an entire dataset of 11,180 cases. We split the dataset into two subsets to develop and validate the model. Specifically, we used 9,578 records (85.7%) collected between 2012 and 2018 as a training set and the remaining 1,602 data (14.3%) collected in 2019 as a test set for model evaluation (Figure 1). The study protocol conformed to the ethical guidelines of the Declaration of Helsinki and was approved by the Institutional Review Board of Seoul National University Hospital (IRB No. H-2004-117-1118). Owing to the retrospective nature of the study, the board waived the requirement for written informed consent.

### 2.2. CCTA image acquisition and analysis

CCTA image acquisition, post-processing, and interpretation were performed according to the guidelines of the Society of Cardiovascular Computed Tomography (20). A 256-detector row scanner (Brilliance iCT 256; Philips Medical Systems Inc., Cleveland, OH, USA) was used to acquire the images with proper quality using either a retrospectively electrocardiography (ECG)-gated or prospectively ECG-triggered protocol, as appropriate. Two level III-equivalent experienced radiologists, who were blinded to the clinical data, assessed, and interpreted all CCTA images. The coronary artery calcium score was measured quantitatively by the sum of the area of coronary calcification using the Agatston scoring system (in units) (21). A coronary atherosclerotic plaque was evaluated in all coronary artery segments with a diameter of ≥2 mm and defined as any distinguishable lesion of >1 mm^2^ within or adjacent to the coronary arterial lumen in at least two independent image planes. The presence, location, and severity of coronary atherosclerotic plaques were evaluated at per-segment and per-patient levels using the modified 15-segment criteria (22).

The presence of obstructive CHD was the main outcome of this study, which was defined as the detection of significant coronary artery stenosis having ≥70% maximal diameter stenosis in any of the four major coronary arteries on CCTA. The severity of maximal coronary stenosis was quantified by visual estimation, with an agreement between two independent radiologists.

### 2.3. Coronary artery stenosis prediction model development

**Figure 2:**
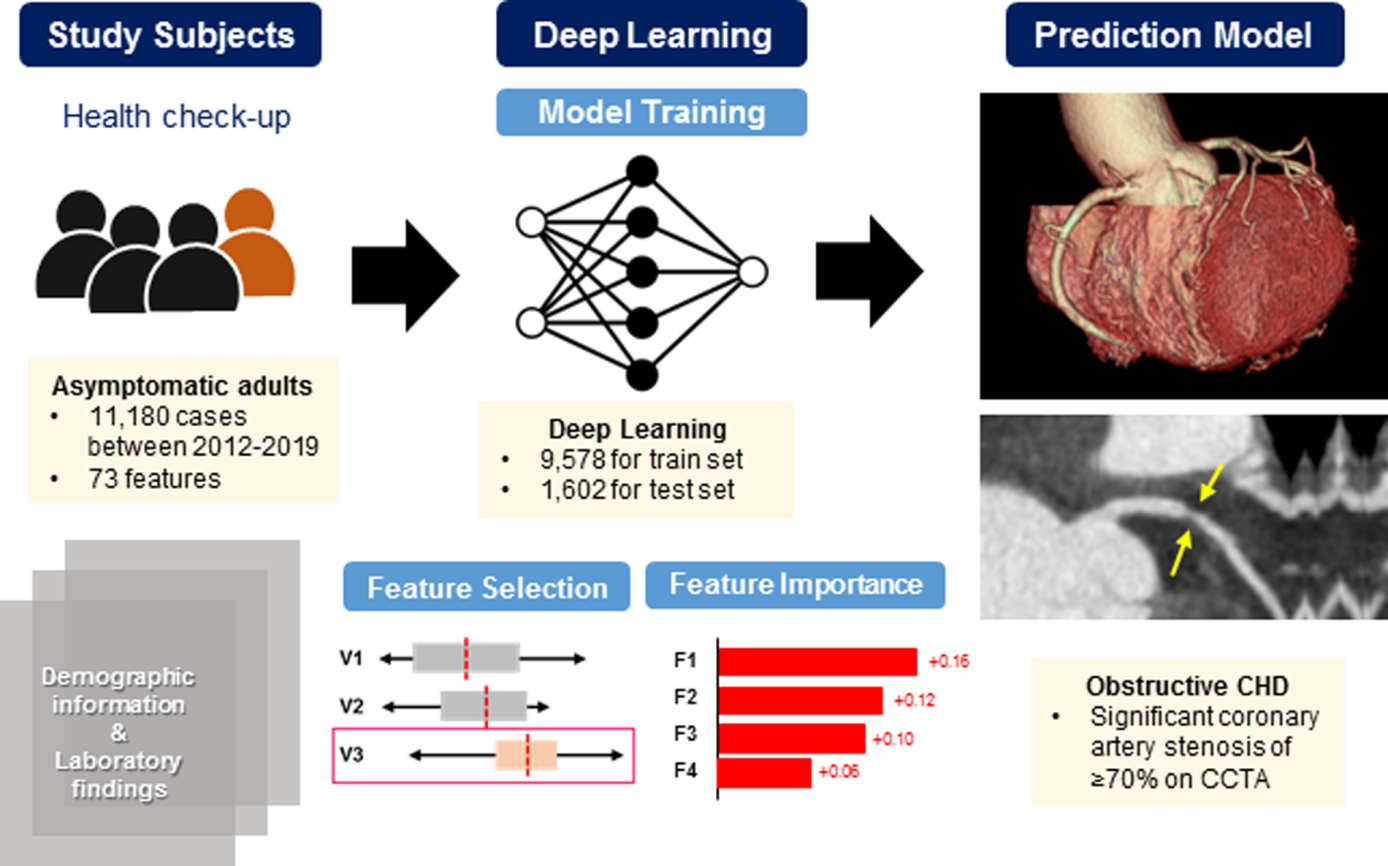
Schematic diagram for the prediction model development. This is a schematic diagram of a newly developed DL-based prediction model for significant coronary artery stenosis on CCTA in 11,180 asymptomatic populations who underwent a routine health check-up. Using various parameters that physicians can readily access in clinical practice, the DL-based model could provide more precise indications of CCTA for the purpose of screening. CCTA, coronary computed tomography angiography; CHD, coronary heart disease; DL, deep learning.

The dataset consists of 11,180 individuals’ medical records including 73 variables. The model development comprised of three steps: (1) data pre-processing, (2) model training and evaluation, and (3) feature importance analysis. The overall process is illustrated in Figure 1, and the key components of this study are presented in Figure 2.

#### 2.3.1. Data pre-processing

The raw format of each record is composed of categorical and numerical variables of different scales with some missing values. The rates of missing values are varied according to the variable types. Every missing value was imputed by the average value of its variable type. To handle with heterogeneous variables, min-max normalization was applied to several categorical variables, and Gaussian normalization was applied to the rest of numerical variables. For several types of numerical variables including age, systolic blood pressure (BP), diastolic BP were discretized using known criteria before normalization The stenosis values (%) were discretized into four categories as follows: diameter stenosis of <30%, 30%–50%, 50%–70%, and ≥70%.

#### 2.3.2. Model training and evaluation

It is well-known that the effect of each variable on stenosis varies. In addition, using all variables in model training may decrease generalization ability, and is not computationally efficient. For these reasons, a stepwise forward/backward feature selection method was used to select more significant variables for stenosis prediction modelling. The final 19 input variables were determined as the sum of 15 variables collected from the feature selection method (Supplementary Table S1) and additional 4 variables known to be clinically important [body mass index (BMI), smoking, hypertension, and diabetes].The feature selection was performed on 12 different randomly split train/validation sets using different random seeds. The feature selection criterion was based on the area under the curve (AUC) calculated from the receiver operating characteristic (ROC) curve of the validation set.

We utilized the DL model to predict the CCTA class based on input features. Our neural network model comprised of two parts. The first part included two locally connected layers among the predefined input variable groups based on prior clinical knowledge, and the second part consisted of two fully connected layers with 512 dimensions. The model’s performance was evaluated based on its ability to classify significant coronary artery stenosis with a threshold of 70%, which was clinically defined as obstructive CHD (23). The models were trained in 2 different settings. The first was a multi-class classification task, where the model directly predicted the class among four categories of diameter stenosis. The second setting involved rearranging the single multiclass classification task into three binary classifications based on the criteria of 30%, 50%, and 70%, which is multi-task learning. The initial learning rate was set to 0.05 and implemented step-wise decay. The activation function used in the model is sigmoid linear unit. We did not use any regularization method in the model.

Currently, to the best of our knowledge, there was no reliable risk prediction model of coronary artery stenosis in apparently healthy asymptomatic populations. Therefore, the model performance was compared with three established clinical scoring systems: coronary artery disease (CAD) consortium scores (24) and the updated Diamond-Forrester (UDF) method (25) as estimates for the pretest probability of obstructive CAD in patients with chest pain and PCE as a well-known tool to estimate the 10-year risk of the clinically relevant endpoint of atherosclerotic cardiovascular disease (26).

Additionally, we implemented conventional machine learning models, including linear regression, random forest, and eXtreme Gradient Boosting (XGBoost), as comparison targets to verify the prediction performance of our neural network model.

#### 2.3.3. Feature importance analysis

Interpreting the reasons behind the model’s decision is crucial, especially for clinical purposes. To enhance the interpretability of the model predictions, we adopted the SHapley Additive ExPlanations (SHAP) (27) analysis after the model training. It enables the deep neural network to be interpretable, providing each variable’s contribution to the model decision. Based on the obtained SHAP values, the relative significance of each variable to the prediction target was identified.

### 2.4. Clinical and laboratory evaluation

Clinical and laboratory data were collected, as published previously (28). Anthropometric measurements were taken by a trained nurse on the day of the health examination. BMI was calculated as weight divided by height in meters (kg/m^2^), and waist circumference (WC) was measured at the midpoint between the lower costal margin and iliac crest. BP and heart rate were taken as average values after 2 measurements using an automated BP monitor with at least 5-min interval in a seated position. To collect information on smoking, alcohol intake, education, monthly income, and personal and family medical histories, a self-reported questionnaire was used. Laboratory data included white blood cell (WBC) count, hemoglobin, serum total cholesterol, high-density lipoprotein (HDL) cholesterol, fasting glucose, glycated hemoglobin (HbA1c), serum albumin, estimated glomerular filtration rate (eGFR), and urine albumin to creatinine ratio levels. An automatic analyzer at the Department of Laboratory Medicine at Seoul National University Hospital (Toshiba 200 FR autoanalyzer; Toshiba, Tokyo, Japan) was used to analyze all laboratory tests.

### 2.5. Statistical analysis

Continuous variables are described as mean ± standard deviation or median (interquartile range) and categorical variables as numbers (%). To compare the differences in baseline characteristics between the study groups, Student’s *t*-test was performed for continuous variables, and Pearson’s chi-square test was applied for categorical variables as required. ROC curves were plotted to identify the predictive power of ML-based models, including our newly developed neural network model, and the comparative scoring systems. The AUC value from each curve was calculated and compared using Bootstrap method with 200 subsampling (29). All statistical analyses were performed using the python, numpy, pandas, and seaborn package of version 3.9, 1.22.3, 1.3.3, and 0.11.2, respectively. A value of two-sided *p *< 0.05 was considered statistically significant.

## 3. Results

### 3.1. Baseline characteristics of the study population

A total of 11,180 cases (mean age, 56.1 years; men 69.8%) were enrolled in this study. The mean BMI and WC were 24.4 kg/m^2^ and 87.5 cm, respectively; approximately 37.6% of the study population was considered obese. On the examination day, systolic and diastolic BP were averaged as 119.7 and 78.5 mmHg, respectively. Conventional cardiovascular risk factors, including hypertension, diabetes, dyslipidemia, and current smoking, were found in 24.8%, 8.5%, 17.3%, and 19.1% of the total subjects, respectively. Approximately one-fifth (21.6%) of the study participants had a family history of premature cardiovascular disease, and 1,341 (12.0%) and 1,933 (17.3%) of them were on anti-platelet agents and statins treatment, respectively. In terms of socioeconomic status, university graduates or higher accounted for 74.0%, and half of the total subjects earned US$8,000 or more per month. The mean values of fasting glucose and HbA1c were 104.9 mg/dl and 5.8%, respectively. Lipid profiles were as follows: total cholesterol 192.5 mg/dl, HDL cholesterol 52.9 mg/dl, LDL cholesterol 115.5 mg/dl, and triglycerides 106.0 mg/dl (median). Subjects in the training set were likely to be older; current smokers; have more comorbidities including hypertension, diabetes, and dyslipidemia; and more educated with higher income than those in the test set. The detailed baseline characteristics of the analyzed cases are presented in Table 1.

**Table 1:** Baseline characteristics of study population.

Variables	Total (*n* = 11,180)	Train set (*n* = 9,578)	Test set (*n* = 1,602)	*p*-value
Demographic information				
Age, years	56.1 ± 8.7	55.8 ± 8.7	57.8 ± 8.8	<0.001
Male sex	7,802 (69.8)	6,682 (69.8)	1,120 (69.9)	0.930
BMI, kg/m^2^	24.4 ± 3.1	24.4 ± 3.1	24.4 ± 3.1	0.455
WC, cm	87.5 ± 8.6	87.3 ± 8.6	89.0 ± 8.7	<0.001
Systolic BP, mmHg	119.7 ± 13.7	119.5 ± 13.7	120.8 ± 14.0	<0.001
Diastolic BP, mmHg	78.5 ± 10.0	78.4 ± 10.0	78.9 ± 10.2	0.048
Heart rate/min	67.5 ± 11.0	67.7 ± 11.1	66.2 ± 10.4	<0.001
Hypertension	2,769 (24.8)	2,282 (23.8)	487 (30.5)	<0.001
Diabetes mellitus	952 (8.5)	754 (7.9)	198 (12.4)	<0.001
Dyslipidaemia	1,933 (17.3)	1,461 (15.3)	472 (29.6)	<0.001
FHx of hypertension	2,810 (25.2)	2,385 (24.9)	425 (26.6)	0.145
FHx of diabetes	2,151 (19.3)	1,830 (19.1)	321 (20.1)	0.348
FHx of CVD	2,413 (21.6)	2,042 (21.3)	371 (23.2)	0.091
Smoking				<0.001
Never smoking	5,448 (48.9)	4,744 (49.6)	704 (45.1)	
Former smoking	3,558 (32.0)	3,089 (32.3)	469 (30.2)	
Current smoking	2,130 (19.1)	1,741 (18.2)	389 (24.9)	
Alcohol consumption				0.030
No drinking	2,363 (22.2)	1,986 (21.8)	377 (24.8)	
Moderate drinking	7,234 (68.0)	6,230 (68.4)	1,004 (66.1)	
Heavy drinking	1,034 (9.7)	895 (9.8)	139 (9.1)	
Education				<0.001
Under middle school	233 (2.3)	209 (2.4)	24 (1.6)	** **
Middle school	287 (2.8)	265 (3.0)	22 (1.4)	** **
High school	1,501 (14.6)	1,292 (14.8)	209 (13.6)	** **
College	5,007 (48.7)	4,260 (48.6)	747 (48.7)	** **
Post-college	3,262 (31.7)	2,732 (31.2)	530 (34.6)	** **
Monthly income, (10,000)KRW*			** **	<0.001
<300	500 (4.4)	449 (5.6)	51 (4.1)	** **
300–500	831 (7.4)	734 (9.1)	97 (7.7)	** **
500–800	1,116 (10.0)	960 (11.9)	156 (12.4)	** **
800–1,000	1,210 (10.8)	1,045 (13.0)	165 (13.2)	** **
1,000–1,500	1,977 (17.7)	1,748 (21.7)	229 (18.3)	** **
1,500–2,000	1,219 (10.9)	1,058 (131.)	161 (12.8)	** **
>2,000	2,469 (22.1)	2,074 (25.7)	395 (31.5)	** **
Anti-platelets	1,341 (12.0)	1,173 (12.3)	168 (10.1)	0.050
Lipid lowering agents	1,933 (17.3)	1,461 (15.3)	472 (29.6)	<0.001
Laboratory findings				
CRP, mg/dl	0.2 ± 0.5	0.2 ± 0.5	0.1 ± 0.2	<0.001
WBC, /ul	5.4 ± 1.5	5.4 ± 1.5	5.5 ± 1.5	0.459
Haemoglobin, mg/dl	14.6 ± 1.3	14.7 ± 1.3	14.5 ± 1.3	<0.001
Fasting glucose, mg/dl	104.9 ± 23.2	104.7 ± 23.6	105.8 ± 21.0	0.103
HbA1c, %	5.8 ± 0.7	5.8 ± 0.8	5.9 ± 0.7	<0.001
Total cholesterol, mg/dl	192.9 ± 37.3	193.5 ± 35.9	189.3 ± 44.6	<0.001
Triglyceride, mg/dl	106.0 (75.0)	106.0 (76.0)	101.0 (71.5)	0.065
LDL cholesterol, mg/dl	115.5 ± 34.5	116.0 ± 32.9	112.5 ± 42.5	<0.001
HDL cholesterol, mg/dl	52.9 ± 13.3	52.9 ± 13.4	53.0 ± 12.3	0.810
Total-HDL cholesterol	140.1 ± 36.7	140.7 ± 35.2	136.4 ± 44.2	<0.001
Albumin, mg/dl	4.4 ± 0.3	4.4 ± 0.3	4.4 ± 0.3	0.075
eGFR, ml/min/1.73 m^2^	90.3 ± 15.2	91.0 ± 15.3	86.2 ± 14.0	<0.001
Urine ACR, mg/g	5.7 ± 1.4	5.7 ± 1.4	5.5 ± 1.29	<0.001

Values are presented as *n* (%), mean ± SD, or median (interquartile range).

ACR, albumin to creatinine ratio; BMI, body mass index; BP, blood pressure; CVD, cardiovascular disease; eGFR, estimated glomerular filtration rate; FHx, family history; HbA1c, glycated haemoglobin; HDL, high-density lipoprotein; SD, standard deviation; WBC, white blood cells; WC, waist circumference.

* 1 US dollar = 1,300 KRW.

### 3.2. Feature selection for DL-based prediction model

**Figure 3:**
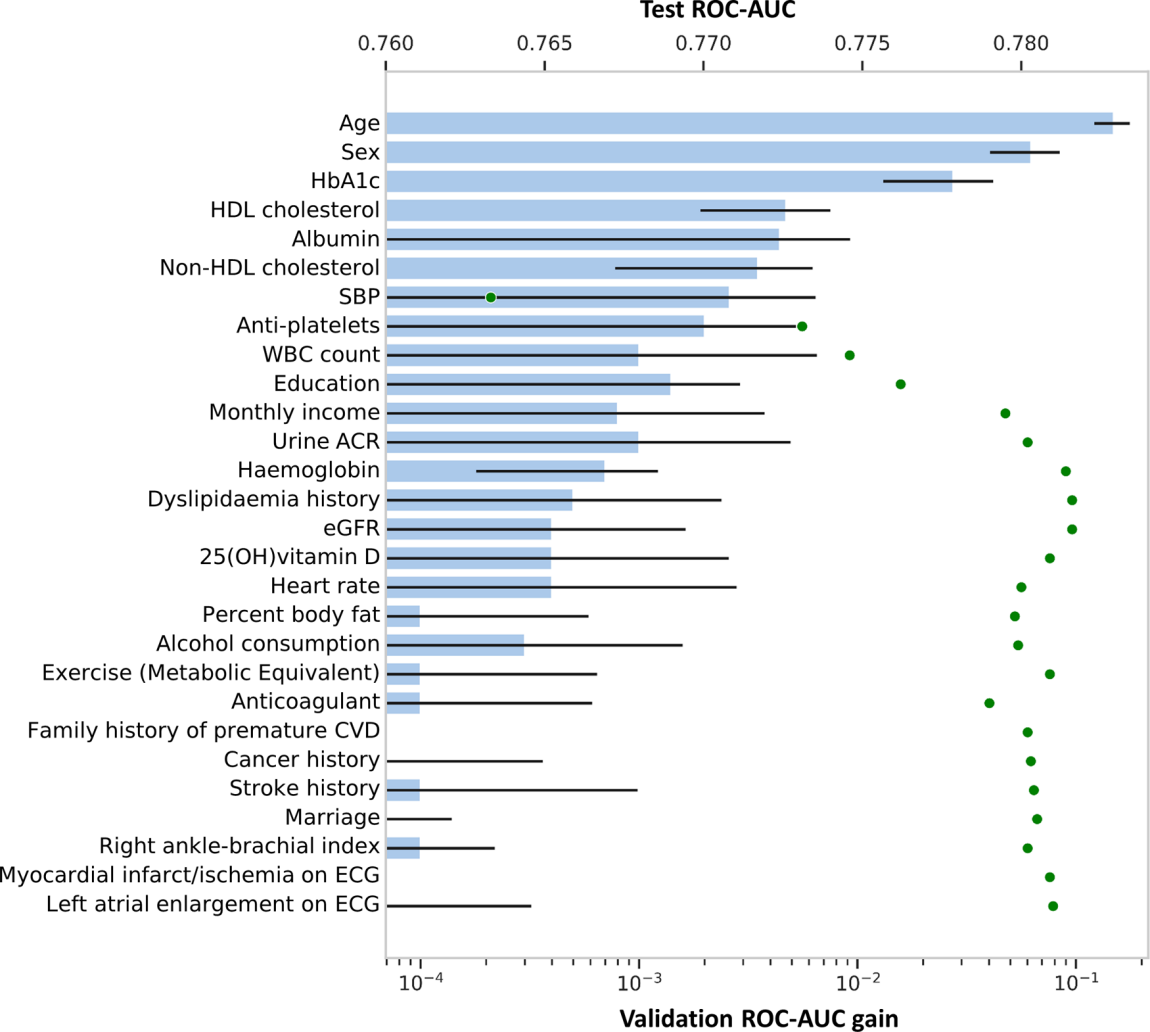
Increase of predictive value upon forward feature selection. Features in Y-axis are arranged by forward selection in order. The bar graph, generated from the validation dataset, shows how performance gains when variables are added to the model, on a logarithmic scale. Error bar shows its 95% confidence interval. Scatter dot plot shows an increase in ROC-AUC of the test dataset when using top-k features, indicating that performance saturates shortly after adding a few variables to the model. CVD, cardiovascular disease; ECG, electrocardiography; eGFR, estimated glomerular filtration rate; HbA1, glycated hemoglobin; HDL, high-density lipoprotein; OH, hydroxy; SBP, systolic blood pressure; WBC, white blood cells; other abbreviations as Figure 1.

Figure 3 illustrates the improvement in prediction performance resulting from forward feature selection using all variables collected from the validation set. Supplementary Table S1 presents the cumulative ROC-AUC with adding variables to model, from top to bottom**.** For the prediction of significant coronary artery stenosis, age, sex, socioeconomic status including education and monthly income level, dyslipidemia, and several laboratory variables including eGFR, hemoglobin, HbA1c, and HDL cholesterol were the highly ranked features. As expected, age and sex were the top 2 predictive features of the DL-based model. Among the conventional cardiovascular risk factors, HbA1c, HDL cholesterol, non-HDL cholesterol, systolic BP, and a history of dyslipidemia were sequentially important in estimating the possibility of obstructive CHD on CCTA. In addition, WBC count and albumin level significantly contributed to the performance of the DL-based model. Interestingly, we observed that personal education and monthly income levels were selected for the DL-based model to predict obstructive CHD. As mentioned above, 4 variables known to be cardiovascular risk factors were added on 15 selected features from forward feature selection method. Therefore, a total of 19 selected variables were used in the neural network model development.

### 3.3. Comparison of prediction models for coronary artery stenosis

**Figure 4:**
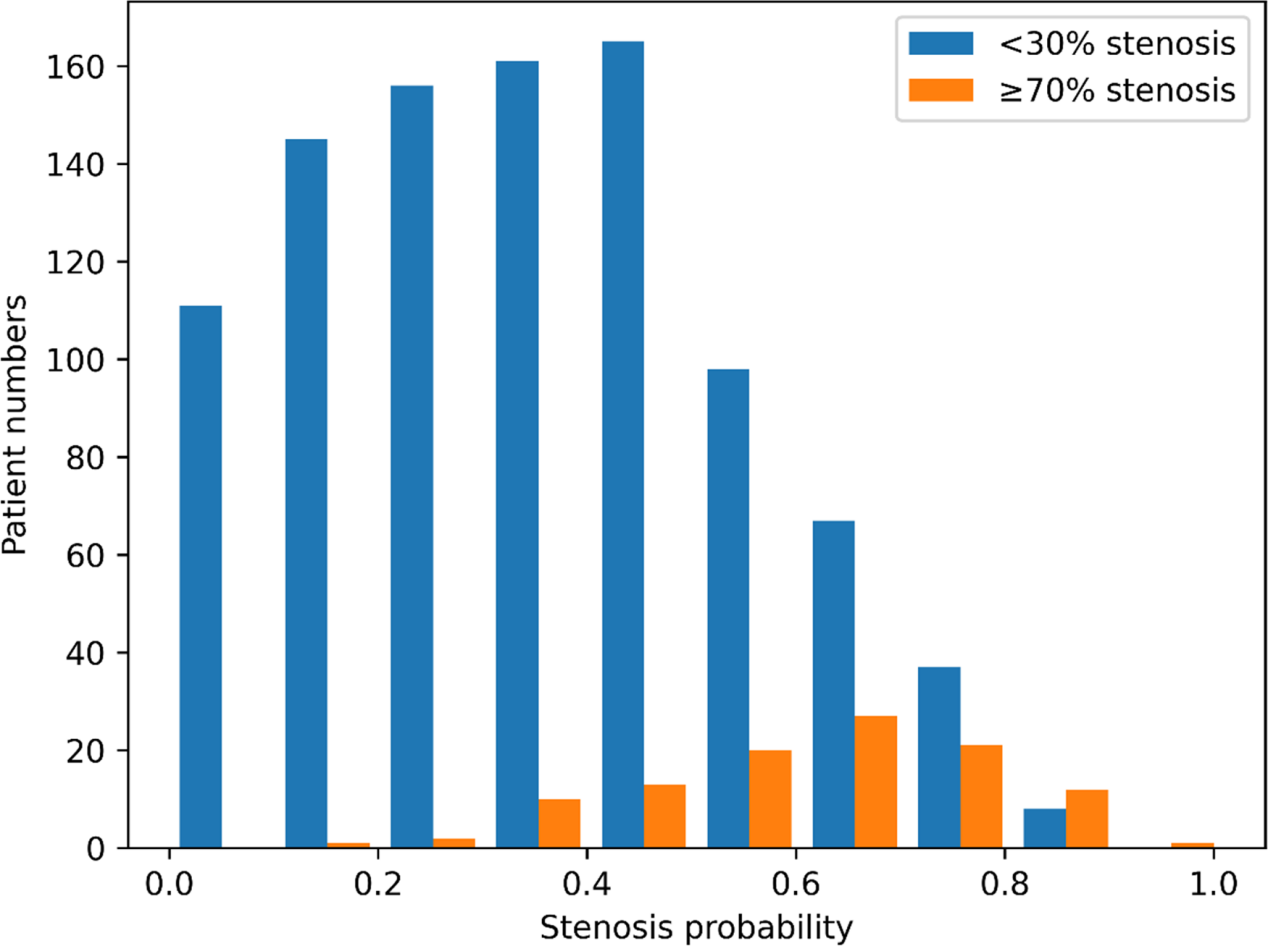
Outcome distribution of individuals with obstructive and non-obstructive CHD. The X-axis represents the probability of coronary artery stenosis, and the Y-axis represents the number of individuals in the test set. The blue and orange bars indicate individuals predicted to have coronary artery stenosis of <30% and ≥70%, respectively.

**Figure 5:**
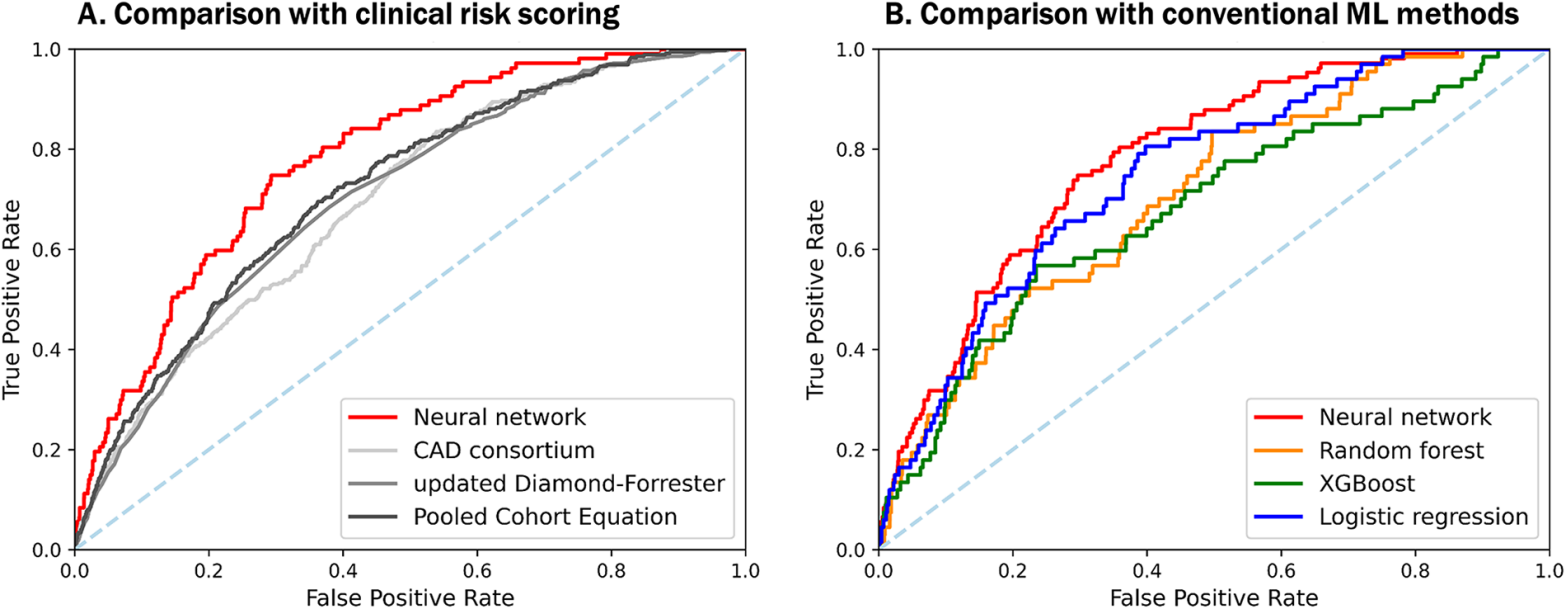
Comparison of predictive performances among a neural network-based model, other DL-based models, and clinical risk scoring models with selected features. Both plots show the ROC-AUC of various models with selected features. (**A**) Our neural network-based prediction model demonstrates significantly better performance than previously known clinical scoring systems, with ROC-AUC of 0.782, even in asymptomatic populations. (**B**) Among the machine learning-based models, a neural network with multi-task shows the best performance to predict significant coronary artery stenosis on CCTA. CAD, coronary artery disease; DL, deep learning; XGBoost, eXtreme gradient boosting; other abbreviations as Figures 1, 2.

Among all the participants, obstructive CHD was observed in 516 (4.6%; 4.2% in training set and 6.8% in test set). Individual prediction scores were calculated in the test set, and the results were expressed as a function of the presence of obstructive CHD, presenting a bimodal score distribution with a higher prevalence of obstructive CHD in subjects with higher scores (Figure 4). When comparing the predictive power of machine learning models, the neural network with multi-task learning produced the better performance to predict significant coronary artery stenosis of ≥70% [AUC 0.782, 95% confidence interval (CI) 0.749–0.820] than Random Forest (AUC 0.695, 95% CI 0.656–0.730), XGBoost (AUC 0.732, 95% CI 0.680–0.788), and logistic regression (AUC 0.749, 95% CI 0.708–0.795) (all *p* < 0.001) (Figure 5A, Supplementary Table S2). This result supported that the neural network-based model for predicting obstructive CHD outperformed the conventional DL methodologies. The sensitivity, specificity, positive predictive value, negative predictive value, and balanced accuracy of the neural network with multi-task learning were 0.757, 0.675, 0.143, 0.975, and 71.6%, respectively.

Compared with PCE (AUC 0.719, 95% CI 0.699–0.743), CAD consortium score (AUC 0.696, 95% CI 0.677–0.717), and UDF scores (AUC 0.705, 95% CI 0.684–0.726), our neural network model had a significantly higher area under the ROC curves for the prediction of obstructive CHD than the clinical scoring systems (all *p* < 0.001) (Figure 5B, Supplementary Table S2).

Furthermore, we trained ML-based models without the feature selection process. The neural network model outperformed the others in this setting as well (Supplementary Figure S1). Notably, the feature selection not only preserved the model’s performance but also improved its generalization to outperform the models trained on all features, as demonstrated by the higher ROC-AUC value.

A further comparative analysis of model predictability was performed, and the correlation between the predicted and actual stenosis ratios was calculated. Each calibration plot of the proposed neural network, CAD consortium, and UDF was shown in Supplementary Figure S2, respectively. The proposed neural network provided robust and accurate predictability in all probability regions, whereas the others showed less correlation, suggesting a better performance in the asymptomatic individuals.

### 3.4. Explainability of the neural network model

**Figure 6:**
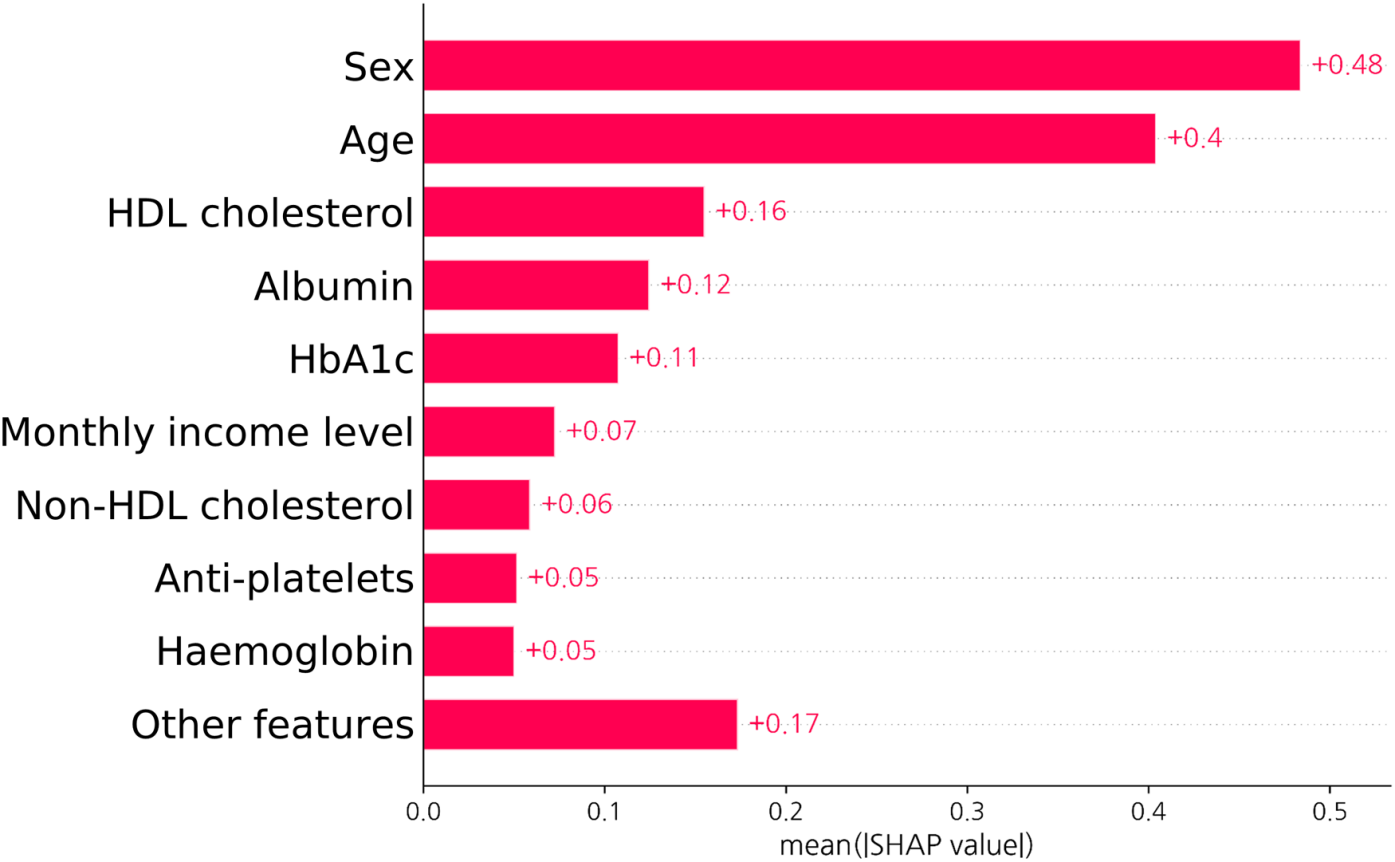
SHAP value of a neural network-based model. The bar graph shows how each feature contributes to the model decision in test set. As the contribution of each input feature is described as an absolute value, the sign of the effect of each variable is not represented. Abbreviations as Figures 1, 3.

Figure 6 depicts the SHAP value of each input feature in predicting the probability of obstructive CHD as obtained from the SHAP analysis. In order of SHAP value, sex, age, and HDL cholesterol levels were the most significant factors in predicting obstructive CHD in asymptomatic individuals.

## 4. Discussion

In the present study including 11,180 apparently healthy asymptomatic adults who underwent CCTA for routine health check-ups, the main findings were as follows: (1) obstructive CHD, defined as significant coronary artery stenosis of ≥70% on CCTA, was found in 4.6% of this asymptomatic population; (2) the DL-based risk prediction model for significant coronary artery stenosis was successfully developed using a multi-task learning with feature selection, which demonstrated superior performance compared to the clinical pretest probabilities and other ML-based models; (3) among the variables that were readily assessed in routine clinical practice, age, sex, education and monthly income level, dyslipidemia, and laboratory variables including eGFR, hemoglobin, HbA1c, and HDL cholesterol were the highly ranked features to predict significant coronary artery stenosis on CCTA.

Although clinicians have bent their best efforts to appropriately manage CHD and improve the prognosis for decades, the global burden of CHD, in terms of disabilities and deaths, continued to increase (1, 30). Early detection and prevention are the most effective ways to reduce the impact of CHD, given the limited healthcare resources (31). A conventional approach in clinical practice is to start validated clinical risk scoring systems, such as PCE or SCORE, and direct downstream testing (3, 4). However, since these probabilistic risk scores were developed in older populations and mainly validated in symptomatic patients for the purpose of predicting major cardiovascular events, including myocardial infarction, stroke, or cardiovascular death, the risk in younger, healthy, and asymptomatic populations is likely to be underestimated and inaccurate (32, 33). The advent of CCTA has provided a paradigm shift to assess cardiovascular risk, particularly in low-risk asymptomatic populations. Many previous studies using CCTA have reported a higher prevalence of silent coronary atherosclerosis in the asymptomatic populations than expected, and further, individuals with obstructive CHD were not uncommon (10, 11). In the SCAPIS cohort, approximately 1 of 3 men and 1 of 4 women were found to have coronary atherosclerosis in the subgroup classified as low risk by PCE or SCORE from the general population (11). In addition, Choi et al. demonstrated that 5% and 2% of asymptomatic adults with a mean age of 50 years had CCTA-derived coronary artery stenosis of ≥50% and ≥75%, respectively (10). However, currently, no reliable prediction model is proven to detect coronary artery stenosis in apparently healthy asymptomatic populations. Hence, there is a need for a clinical method to easily screen obstructive CHD candidates from low-risk asymptomatic groups that would be missed under the traditional approach.

In this study, given the insufficient clinical methodology for predicting risk in the asymptomatic population, we utilized the DL-based method and successfully developed the risk prediction neural network model to detect significant coronary artery stenosis of ≥70% on CCTA in the self-referred apparently healthy asymptomatic population. Our model provided balanced accuracy of 71.6% and achieved an AUC value of 0.782 for the test set, demonstrating good performance and reproducibility. Obviously, ML or DL-based risk prediction models with excellent performance in various study populations have been presented before (17, 34–38). However, previous studies have mainly used ML or DL to predict the prognosis in patients with symptomatic or established cardiovascular disease, such as those with suspected or known CAD, those with acute coronary syndrome, and those who underwent coronary angiography or coronary artery bypass grafting (CABG). In the CONFIRM registry, an ML-based model including clinical parameters and CACS estimated the risk of obstructive CAD on CCTA in suspected CAD patients (17). More recently, the risk of post-CABG mortality was successfully estimated with acceptable performance using various ML models, in 16,850 patients who underwent isolated CABG (36). Although a few studies were performed in the asymptomatic healthy populations, they used only CACS, a simpler tool for estimating total atherosclerotic burden (39, 40). The current study successfully developed the DL-based model in predicting those who are likely to have significant coronary artery stenosis on CCTA, using clinical parameters that are obtained from routine health check-ups among the asymptomatic, apparently healthy individuals. Indeed, this allows the potential to detect CAD early and improve prognosis, considering that CCTA is emerging as a frontline test for the diagnosis of CAD.

Our DL-based risk prediction model included conventional risk factors such as age, sex, systolic BP, HbA1c, and lipid-related variables as well as unfamiliar risk factors such as serum albumin level, WBC count, and socioeconomic status. These are variables that clinicians rarely pay attention to when evaluating whether an individual has obstructive CHD. However, considering that WBC count (41) and serum albumin level (42) were once noted for a close link with CHD, our results were able to bring about a re-perception of solid but overlooked risk factors using the DL method. This is quite consistent with the previous study (43) in that our model was developed using the clinical features that can be readily found in electronic health records in clinical practice. In addition, recent studies have demonstrated that the integration of socioeconomic status into traditional risk factors can allow better risk stratification and prognosis for individuals at risk (44). More noticeably, our DL-based model provided better predictive power than the CAD consortium score, UDF, and other well-known probabilistic risk scores, indicating that the group-specific risk prediction model should be newly established in the low-risk asymptomatic population.

Among the various ML methods available, we chose a DL-based approach to develop our risk prediction model. DL-based methods offer several advantages over conventional approaches in terms of flexibility and generality (45). Traditional statistical methods such as simple linear regression assume that dependent variables should be normally distributed and independent of each other with homoscedasticity (46). Furthermore, the errors obtained from regression analysis should be uncorrelated and constant (47). These constraints raise the practical issues when analyzing heterogeneous real-world data. However, DL-based methods are relatively free of these constraints regarding the distribution of variables. Owing to their applicability, DL-based methods have shown prominent outcomes in various real-world clinical studies in recent times.

In order to improve the interpretability of the proposed risk prediction model, we introduced two additional methods. First, we implemented a forward/backward feature selection during the model training, which resulted in the exclusion of several variables that had relatively less contribution to prediction of significant coronary artery stenosis, thus ensuring the generalization of the model. Second, we conducted SHAP analysis on the prediction results of the test set to analyze the relative significance of each variable. Our findings revealed that sex and age were the most significant factors affecting the degree of coronary artery stenosis on CCTA, which is in accordance with previous studies (12, 24, 25, 27). As previously mentioned, the dataset utilized in this study was collected from the general population and contained both categorical and numerical features. Our neural network outperformed other conventional methods, verifying the superiority and robustness of DL-based methods for analyzing complex data distributions in the real world. In addition, SHAP analysis allows clinicians to identify the specific features of patient data that the model considers to be crucial in the prediction of stenosis. Finally, our model exhibited superior prediction performance on both the test and training sets compared to previous models. Based on these observations, our neural network could alleviate two common limitations of DL-based methods: the lack of explainability (black-box) and loss of generality (overfitting) (48).

This study has some limitations noteworthy to mention. First, this was a single-center single-ethnicity retrospective observational cohort study comprising apparently healthy self-referred asymptomatic individuals. Thus, it may cause selection and referral bias that limits the generalizability of the model. However, we demonstrated that the prevalence of CCTA-derived obstructive CHD was not trivial even in the lower-risk asymptomatic group by applying the DL-based model composed of familiar clinical variables. It is possible to help identify who could benefit from the use of CCTA as a screening tool and prevent the overuse of diagnostic imaging modalities. Obviously, our DL-based risk prediction model is an aspect of tailored medicine that is expected to improve an individual’s prognosis. Further multi-ethnic prospective studies are required to validate our results. Second, the study endpoint was the presence of obstructive CHD, defined as ≥70% maximal diameter stenosis in coronary arteries on CCTA, which was assessed by visual estimation. Accordingly, the percentage of stenosis may be overestimated, especially in cases with severe coronary artery calcification or motion artifacts. To minimize this limitation, all CCTA images were independently analyzed by two level-III experienced radiologists blinded to the clinical information, with full agreement. Additional validation using volumetric measurements is required in future studies. Third, this study used the full routine health check-up dataset, including many clinical, laboratory, and imaging variables. External validation was not performed, because it was difficult to find other large-scale independent cohorts involving a full dataset, which might have led to overfitting. Further studies in other populations should be considered to validate the current model and extend the indications. Lastly, as shown in Table 1, the mean values of several variables were significantly different between the train and test datasets. This situation could be problematic because it may lead to poor generalizability of the model. However, despite the different characteristics, our model overcame the gap and achieved robust performances on both sets.

## 5. Conclusions

In conclusion, in a cohort comprising 11,180 apparently healthy asymptomatic adults, we successfully developed a DL-based risk prediction model for detecting CCTA-derived obstructive CHD with acceptable accuracy. Our novel model showed better predictive performance than previous well-known risk scoring systems, suggesting more precise indications for CCTA as a screening tool for further risk stratification in asymptomatic populations. Therefore, the utilization of this DL-based model may help clinicians make medical decisions in terms of early diagnosis and primary prevention, and promote the cardiovascular health of individuals.

## Data Availability

The datasets presented in this article are not readily available. Requests to access the datasets should be directed to the corresponding author [MJK], upon reasonable request.
