# Supplementary material for: Deep learning-based prediction for significant coronary artery stenosis on coronary computed tomography angiography in asymptomatic populations
Source: Front Cardiovasc Med. 2023 Jun 21;10:1167468. doi: 10.3389/fcvm.2023.1167468 (PMC10320158; doi:10.3389/fcvm.2023.1167468)
Supplement: Supplementary file 1 [file Table1.docx]

**Supplementary Table S1. The cumulative increase of the prediction performance (from top to bottom)**

| **Selected Features** | **The cumulative ROC-AUC** |
| --- | --- |
| Age | 0.6548 |
| Sex | 0.7236 |
| HDL cholesterol | 0.7515 |
| HbA1c | 0.7519 |
| Total cholesterol – HDL cholesterol | 0.7578 |
| Albumin | 0.7583 |
| Systolic BP | 0.7633 |
| Anti-platelets | 0.7731 |
| WBC | 0.7746 |
| Education | 0.7762 |
| Monthly income | 0.7795 |
| Urine ACR | 0.7802 |
| Haemoglobin | 0.7814 |
| Dyslipidaemia history | 0.7816 |
| eGFR | 0.7816 |

ACR, albumin to creatinine ratio; AUC, area under the curve; BP, blood pressure; eGFR, estimated glomerular filtration rate; HbA1c, glycated haemoglobin; ROC, receiver operating characteristics; WBC, white blood cell.

**Supplementary Table S2. Comparison of predictive power among various models**

|  | **AUC (95% CI)** | ***p*** |
| --- | --- | --- |
| ***Machine learning based models*** | | |
| Neural network | 0.782 (0.749 – 0.820) | (reference) |
| Logistic regression | 0.749 (0.708 – 0.795) | < 0.001 |
| Random forest | 0.703 (0.652 – 0.759) | < 0.001 |
| XGBoost | 0.732 (0.680 – 0.788) | < 0.001 |
| ***Clinical scoring systems*** |  |  |
| PCE | 0.719 (0.699 – 0.743) | < 0.001 |
| CAD consortium score | 0.696 (0.677 – 0.717) | < 0.001 |
| UDF score | 0.705 (0.684 – 0.726) | < 0.001 |

The predictive power of all the models was compared with that of neural network-based model. CAD, coronary artery disease; DL, deep learning; PCE, Pooled Cohort Equation; UDF, updated Diamond-Forrester; XGBoost, eXtreme Gradient Boosting.

**Supplementary Figure S1. Comparison of predicted performance among DL-based models with total features**


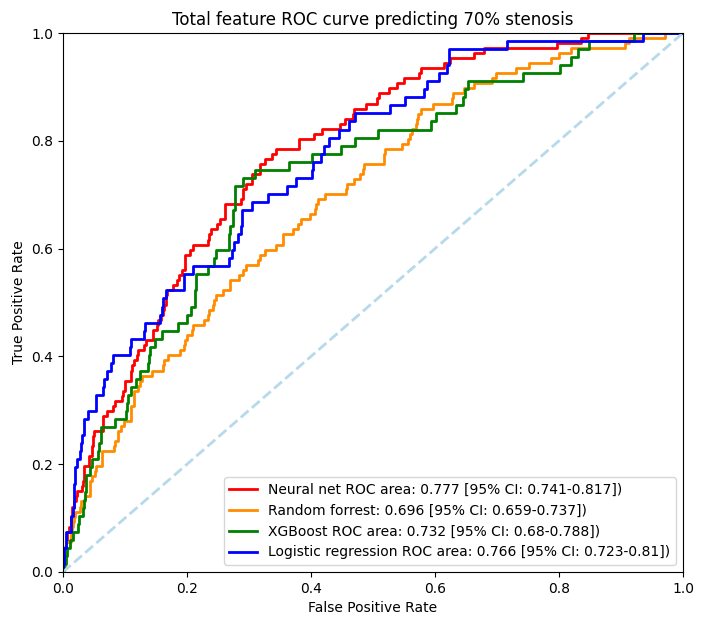


This plot shows ROC-AUC of various DL-based models with total features. Model with total features shows a similar trend with selected feature models. AUC, area under the curve; ROC, receiver operating characteristic.

**Supplementary Figure S2. Calibration plot of model prediction**


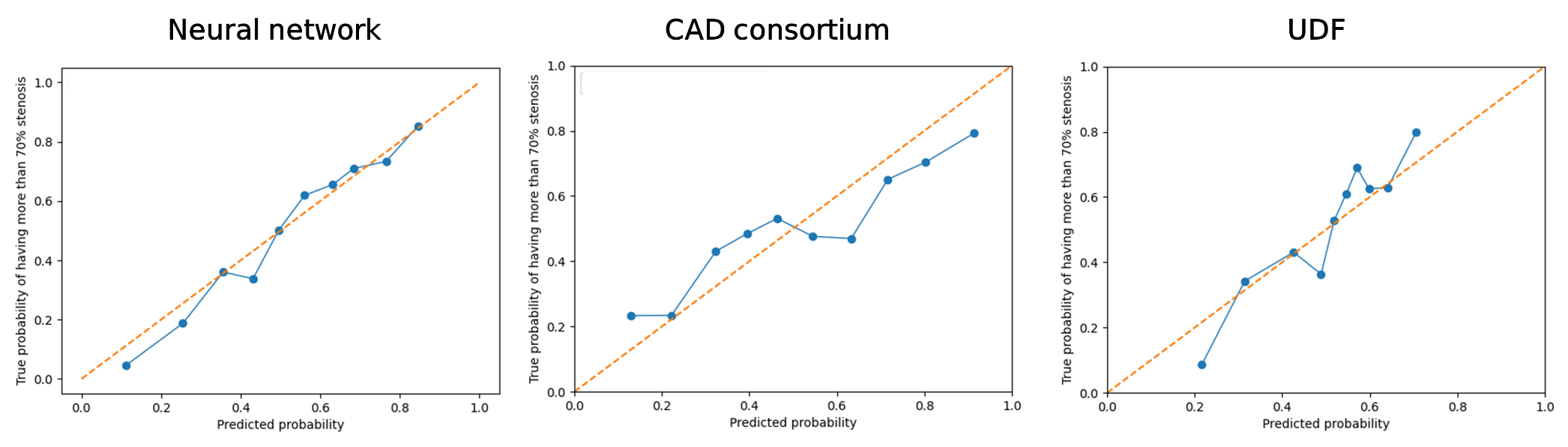


The calibration plot shows a correlation between the model prediction of having ≥70% stenosis and the actual ratio of having ≥70% stenosis. UDF and CAD consortium scores have been reconstructed based on their paper. Since the original UDF and CAD consortium showed shifted calibration plot due to the change in the study population and stenosis patient ratio, we fit the model by adjusting the intercept. Also, for all plots, we balanced the number of patients with and without stenosis using oversampling. CAD, coronary artery disease; UDF, updated Diamond-Forrester.
